# High-Quality Draft Whole-Genome Sequences of Three Strains of *Enterobacter* Isolated from Jamaican *Dioscorea cayenensis* (Yellow Yam)

**DOI:** 10.1128/genomeA.00170-14

**Published:** 2014-03-13

**Authors:** Han Ming Gan, Alexander J. Triassi, Matthew S. Wheatley, Michael A. Savka, André O. Hudson

**Affiliations:** a School of Science, Monash University Malaysia, Jalan Lagoon Selatan, Bandar Sunway, Selangor, Malaysia; b The Thomas H. Gosnell School of Life Sciences, Rochester Institute of Technology, Rochester, New York, USA

## Abstract

Here we report the whole-genome sequences of three endophytic bacteria, *Enterobacter* sp. strain DC1, *Enterobacter* sp. strain DC3, and *Enterobacter* sp. strain DC4, from root tubers of the yellow yam plant, *Dioscorea cayenensis*. Preliminary analyses suggest that the genomes of the three bacteria contain genes involved in acetoin and indole-3-acetic acid metabolism.

## GENOME ANNOUNCEMENT

The plant *Dioscorea cayenensis*, commonly referred to as “yellow yam,” is a staple crop in Jamaica. The edible root tubers are agriculturally, economically, and culturally important (1). Jamaican farmers produced ~0.13 million metric tons of yam in 2011 (http://faostat.fao.org/site/339/default.aspx). In addition to being a source of compounds that support growth and development, such as thiamine, riboflavin, niacin, folate, and amino acids, the plant accumulates the secondary metabolite compounds polyphenols and tannins, among others, which are used in a range of applications (2). To gain some insights into the bacteria that associate with *D. cayenensis*, we embarked on a project which resulted in the identification of three bacteria from the genus *Enterobacter*. Plant-associated bacteria that are beneficial to *D. cayenensis* have the potential to improve crop production. The sequenced bacteria were isolated from *D. cayenensis* grown in the Westmoreland Parish of Jamaica.

Genomic DNA was extracted from the *Enterobacter* sp. strains using the GenElute bacterial genomic kit (Sigma-Aldrich, St. Louis, MO) and prepared for whole-genome sequencing using a Nextera XT library preparation kit (Illumina, San Diego, CA). Whole-genome sequencing was performed using the Illumina Miseq (250-bp paired-end reads). The reads were error corrected and assembled *de novo* using Spades (3). The generated contigs were subsequently scaffolded and gap closed using SSPACE and GapFiller, respectively (4, 5). Genome annotation was performed using Prokka (http://www.vicbioinformatics.com/software.prokka.shtml), which incorporated Prodigal 2.60, Aragorn, and RNAmmer 1.2 for the prediction of open reading frames (ORFs), tRNAs, and rRNAs, respectively (6–8). Further annotation augmentation was performed using InterProScan5 (9). The *de novo* assembly resulted in assemblies of 4,590,638 bp (95× coverage, GC content of 56.0%, *N*_50_ of 641,161 bp, and 18 contigs), 5,076,304 bp (90× coverage, GC content of 55.7%, *N*_50_ of 436,370 bp, and 34 contigs), and 5,211,711 bp (90× coverage, GC content of 55.6%, *N*_50_ of 553,993 bp, and 30 contigs) for strains DC1, DC3, and DC4, respectively. Strain DC1 contains 4,275 ORFs, 79 tRNAs, and 11 rRNAs; strain DC3, 4,727 ORFs, 79 tRNAs, and 11 rRNAs; and strain DC4, 4,857 ORFs, 83 tRNAs, and 10 rRNAs.

Similar to those of *Enterobacter* sp. strain SST3 and *Enterobacter* sp. strain 638, the genomes of strains DC1, DC3, and DC4 lack the *nif* genes necessary to facilitate nitrogen fixation (10, 11). The lack of *nif* genes in the genomes of the bacteria may indicate a different mechanism(s) of symbiotic and/or synergistic interactions with the host plant. Genomic analyses revealed the presence of a *budABC* gene cluster involved in the production of acetoin and 2,3-butanediol. Acetoin and 2,3-butanediol are known to stimulate plant growth via the activation of the cytokinin-signaling pathways (12). The gene *ipdC*, which encodes indole-3-pyruvate decarboxylase, was identified in all three strains, suggesting a role in the biosynthesis of the plant growth hormone indole-3-acetic acid.

### Nucleotide sequence accession numbers.

Whole-genome shotgun projects for *Enterobacter* sp. strain DC1, *Enterobacter* sp. strain DC3 and *Enterobacter* sp. strain DC4 have been deposited at DDBJ/EMBL/GenBank under the accession numbers AZUA00000000, AZXZ00000000, and AZUB00000000, respectively.
